# Conserved LIR-specific interaction of Sigma-1 receptor and GABARAP

**DOI:** 10.1016/j.isci.2025.113287

**Published:** 2025-08-05

**Authors:** Marius Wilhelm Baeken, Maximilian Christ, Daniel Schmitt, Wencke Trein, Heike Nagel, Albrecht Martin Clement, Hagen Körschgen, Christian Behl

**Affiliations:** 1 Institute of Pathobiochemistry, The Autophagy Lab, University Medical Center of the Johannes Gutenberg-University Mainz, Duesbergweg 6, 55128 Mainz, Germany

**Keywords:** Biochemistry, Molecular biology

## Abstract

Among its various functions, the sigma-1 receptor (σ1R) has been reported to modulate macroautophagy. It is currently unknown how this activity is mediated. We phylogenetically, structurally, and biochemically analyzed σ1R regarding its function in autophagy. We identified several putative LC3-interacting-regions (LIRs) that may mediate interactions with ATG8 proteins, which are known to promote autophagosome biogenesis, autophagic cargo reception, and lysosome fusion. Human σ1R comprises a LIR motif (hLIR5) typical for interaction with a specific ATG8, GABARAP. Biochemically, we uncovered a GABARAP-σ1R interaction depending on this motif via peptide array analysis and confirmed this via immunoprecipitation, co-localization, and proximity ligation assays. In addition, we verified a LIR-dependent presence of σ1R in isolated native autophagic vesicles. Excitingly, two point mutations within this LIR that have previously been reported to be associated with autosomal-recessive distal spinal muscular atrophy lack the ability to interact with GABARAP, highlighting the physiological relevance of the hLIR5-mediated σ1R-GABARAP interaction.

## Highlights

• Sigma-1 receptor (σ1R) interacts in a conserved canonical LIR-mediated fashion with GABARAP
• σ1R-GABARAP interaction is induced upon activation by σ1R agonists
• LIR motif links σ1R to autophagic vesicles

## Graphical abstract

**Figure:**
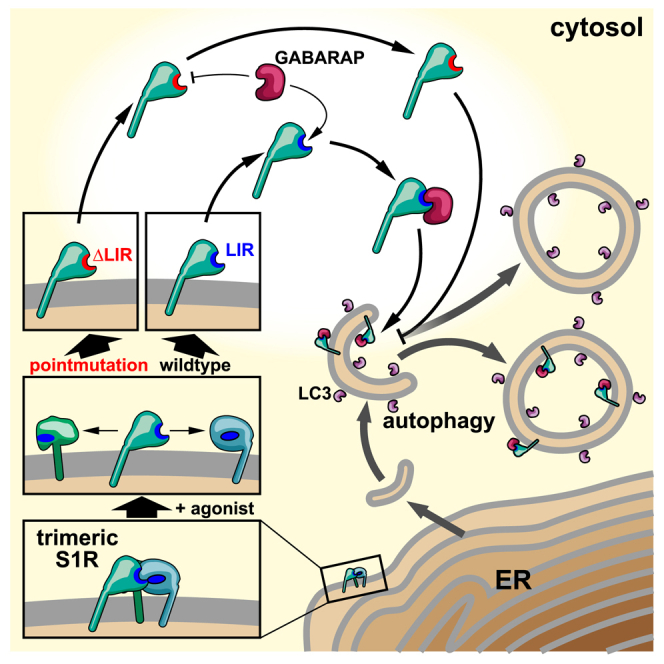

## Introduction

The sigma-1 receptor (σ1R), originally classified as a subclass of opioid receptors,^1^ together with the sigma-2 receptor, has been recognized as its own class of unique receptors.^2^ While it does not display homology to any other mammalian receptor, σ1R is strongly conserved in mammals.^3^^,^^4^^,^^5^ The receptor primarily localizes at the mitochondria-associated membrane (MAM) of the endoplasmic reticulum (ER). Interestingly, upon activation by specific ligands, σ1R translocates to various cellular compartments such as the nuclear envelope, the mitochondrial outer membrane, and the plasma membrane.^6^^,^^7^^,^^8^^,^^9^^,^^10^

The crystal structure of human σ1R has revealed a trimeric organization, which is considered to be the protein’s inactive state.^11^ Several studies have evidenced physiological changes in oligomerization driven by ligand binding.^11^^,^^12^^,^^13^^,^^14^^,^^15^ However, a uniform classification of σ1R ligands as agonists or antagonists is challenging. Based on a set of structural and functional studies, the currently accepted mode of action points toward a stabilization of oligomeric structures by antagonists and a monomerization of σ1R upon activation or agonist exposure.^14^^,^^16^^,^^17^^,^^18^^,^^19^ However, given that certain agonists deviate from this model, this view might be too simplistic.^20^

Canonically, σ1R interacts with and modulates the activities of voltage-gated potassium channels, opioid receptors, dopamine receptors, and signaling molecule receptors,^21^^,^^22^^,^^23^^,^^24^^,^^25^ thus strongly affecting cellular homeostasis, neuronal excitability, and neurotransmission. Accordingly, various neuroprotective activities have been shown for σ1R.^26^ Reduced σ1R expression or activity has been linked, among others, to various neurodegenerative diseases, turning σ1R and its activation into a prominent target for therapy and prevention.^27^^,^^28^^,^^29^^,^^30^ Consequently, the activation of σ1R by selective agonists can mediate neuroprotective effects.^31^^,^^32^ One proposed protective mechanism is the stabilization of neuronal protein homeostasis upon σ1R activation.^26^ In line with data by other groups,^33^^,^^34^^,^^35^^,^^36^^,^^37^ we have previously demonstrated that this positive effect might be attributed to enhanced macroautophagy via σ1R activation.^38^ More specifically, σ1R appears to mediate autophagosome lysosome fusion, at least as far as mitophagy is concerned.^37^

Macroautophagy (hereafter referred to as autophagy) is a highly conserved and dynamic lysosomal degradation and clearance pathway for misfolded proteins and deficient organelles. It includes the formation of double-membrane vesicles, called autophagosomes, the sequestration of cargo and the delivery to lysosomes for degradation. Key components of the autophagy process are ubiquitin-like proteins of the ATG8 protein family.^39^ In mammals, these include two protein subfamilies: the LC3 (Microtubule-associated proteins 1A/1B light chain 3) family (LC3A, LC3B, LC3C) and the GABARAP (GABA type A receptor-associated protein) family (GABARAP, GABARAPL1 and GABARAPL2).^39^^,^^40^ They share a primarily cytosolic localization, which changes to a membrane-bound form, both at the inner and outer autophagosomal membrane, upon lipidation.^41^ LC3B is commonly used as a biochemical marker for autophagosomes and as an indicator of the autophagic flux. The fact that many selective autophagy receptors and autophagy adaptors preferentially bind to members of specific ATG8 subfamilies suggests a distinct, non-overlapping function of LC3 and GABARAP proteins.^42^ LC3s are mainly responsible for autophagosome membrane elongation and the transport to lysosomes, as well as binding to selective autophagy receptors.^43^^,^^44^ GABARAPs, on the other hand, were attributed to be more involved in the fusion of autophagosomes with lysosomes.^45^^,^^46^ A recently reported interaction of σ1R with cytoplasmic mRNA of LC3B also links σ1R to autophagy.^47^

The binding of autophagy adaptors or receptors to ATG8 proteins is usually mediated by the LC3 interacting region (LIR), which interact with the LIR docking site (LDS) of the ATG8 proteins. The canonical LIR motif consists of the conserved consensus core sequence [W/F/Y]_Θ_-X_1_-X_2_-[L/V/I]_Γ_ (amino acids in single-letter code, X for a variable amino acid).^48^ In addition, the affinity of this interaction is enhanced by acidic or phosphorylated amino acids at the N- or C-terminus of the consensus sequence.^42^^,^^44^^,^^49^ Specifically for GABARAP, this motif is even more conserved (GABARAP interaction motif, GIM): [W/F]_Θ_ -[V/I]_1_-X_2_-V_Γ_.^50^

Based on the central function and importance of ATG8 proteins in cargo acceptance and transfer to lysosomes, as well as the induction of autophagy by σ1R ligands, we investigated a potential interaction of σ1R with ATG8 proteins in detail to understand its mechanistic link to autophagy.

## Results

### Sequence analysis of human σ1R discloses potential ATG8 interaction sites

Given our previous findings regarding the induction of autophagy by the pharmacological activation of σ1R,^38^ we aimed to further unravel the mechanism behind this regulation. Based on the pivotal position of ATG8 proteins within the autophagic pathway,^51^ we analyzed human σ1R for potential LIR motifs. Indeed, we discovered nine putative candidate sequences sharing the common denominator for canonical LIR motifs [W/F/Y]_Θ_-X_1_-X_2_-[L/V/I]_Γ_ (Table 1). Our previous data on the different functions and specificities of LC3 proteins might suggest specific motif preferences of each human ATG8 paralog.^52^ Therefore, we estimated which human ATG8 paralog might have the highest selectivity toward each putative LIR (Table 1). Interestingly, these studies revealed a potential preference for proteins of the GABARAP-subfamily to bind to the candidate motifs.

**Table 1:** Putative LIR motifs in human σ1R

Nr.	Put. LIR-Sequence (X_0_-X_3_)	Start	End	N-terminal AA (X_-5_-X_-1_)	C-terminal AA (X_4_-X_10_)	Predicted interacting ATG8
1	**W**AA**L**	11	14	GRRWA	LLAVAAV	GABARAP
2	**W**LW**L**	27	30	LTQVV	GTQSFVF	GABARAP
3	**Y**AG**L**	48	51	QLARQ	DHELAFS	GABARAPL2
4	**F**SR**L**	57	60	DHELA	IVELRRL	GABARAP
5	**W**VF**V**	81	84	DEELQ	NAGGWMG	GABARAP
6	**Y**VL**L**	103	107	ASLSE	FGTALGS	LC3B
7	**W**AE**I**	121	124	HSGRY	SDTIISG	LC3C
8	**F**LT**L**	196	199	FSTQD	FYTLRSY	GABARAPL1
9	**F**YT**L**	200	203	DFLTL	RSYARGL	GABARAPL1

Conserved amino acids in single letter code at position X_0_ and X_3_ are marked in bold. Acidic (D/E) residues or potential phosphorylation targets (S/T) in the flanking regions are underlined.

### Phylogenetic and sequence analyses reveal the conservation of hLIR4 to hLIR7

**Figure 1:**
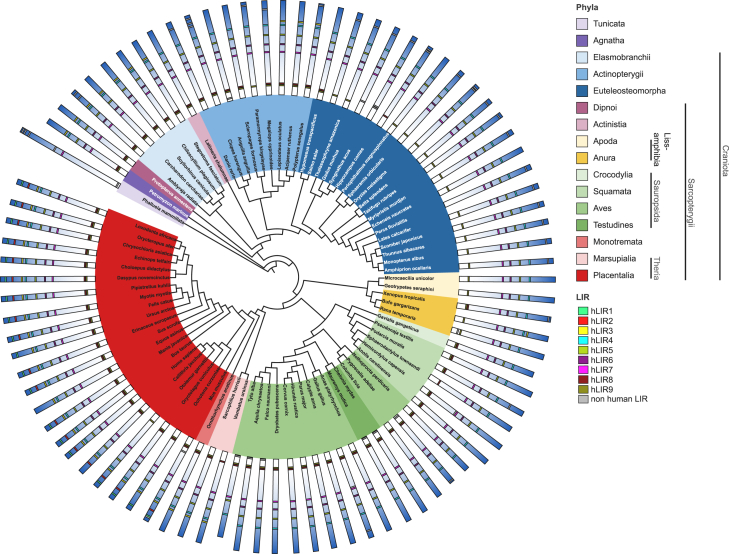
σ1R phylogeny Phylogenetic tree based on the amino acid sequences of σ1R in the *Deuterostomia* using the tunicate *Phallusia mammillata* as outgroup. Major vertebrate phyla are annotated with a specified color code. Schematic structures of corresponding σ1R orthologs are aligned at the tip of each branch. Homologous putative LIR motifs are highlighted within the structure. Putative LIRs corresponding to those of human σ1R are highlighted by color code.

Recently, we successfully employed a phylogeny-driven approach to appraise putative LIR motifs in the human BAG3 protein.^53^^,^^54^ Therefore, we performed a similar analysis for σ1R to narrow down the list of LIR candidates. First, the phylogeny of σ1R in the basal *Gnathostomata* did not reflect the sister-group relationship of *Actinistia* and *Dipnoi* (Figure 1). They are separated by the *Elasmobranchii* and unexpectedly cluster together with the *Actinopterygii*. Thus, the *Sarcopterygii* σ1R appears paraphyletic. In addition, within the amniotes, the basal *Sauropsida* σ1Rs of the analyzed *Testudines* cluster within the *Aves* (Figure 1). Whether the differences in the evolution of the *Craniota* are merely artifacts due to the very high conservation of σ1R or represent selective events might be clarified by further bioinformatic or genomic analyses. Interestingly, the tree displays two phyla discernible into obligate aquatic animals and terrestrial or secondary aquatic animals. Besides this phylogenetic observation, this may functionally be related to σ1R’s role in potassium and calcium signaling, which may differ greatly between these groups.^55^

We used the tunicate *Phallusia mammillata* as an outgroup for our analysis, as it was the most basal ortholog within the *Deuterostomia* branch in the databases. Here, only the homolog to human LIR7 was present. The *Phallusia* σ1R has lost LIR motifs of more basal orthologs and features unique putative LIR motifs in its terminal regions. Within the early vertebrates, σ1R orthologs already feature LIR motifs homologous to hLIR3, 4, 5, 6, 8, and 9. Within the higher *Actinopterygii*, the *Lissamphibia* (except for the toads), and the *Sauropsida,* hLIR3 was lost again. Most of the *Euteleostomorpha* and *Lissamphibia* lost hLIR9. The N-terminal hLIR1 and hLIR2 are innovations of the *Theria* phylum, which also occasionally disappear.

**Figure 2:**
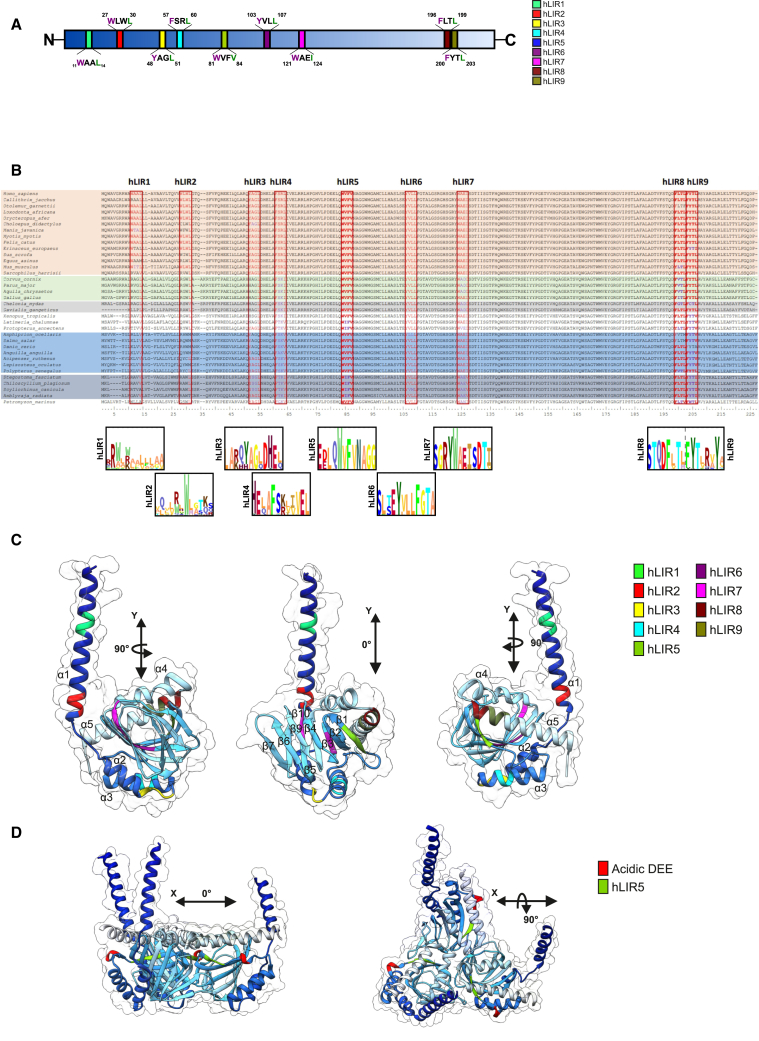
In-depth analysis of LIR conservation and structural accessibility in σ1R (A) Diagram showing putative LIR motifs, their position, and their sequences in human σ1R. (B) Multiple sequence alignment of selected vertebrates shown in Figure 1. Representatives of major phyla are marked by specific background colors (mammals (red), birds (green), reptiles (gray), osteichthyes (blue), and chondrichthyes (dark gray)). Putative human LIRs (hLIR) and their conservation are highlighted in red boxes. Frequency plots of all hLIRs were calculated using skylign to visualize conservation. (C) Rotation of the monomeric human σ1R, modeled using AlphaFold2.^56^^,^^57^ Putative LIRs are highlighted by color code. (D) Crystal structure of the trimeric state of human σ1R (PDB:5HK2). Predicted hLIR5 and N-terminal acidic residues are highlighted.

To elaborate the precise conservation of the motifs (Figure 2A), we generated frequency plots (Figure 2B). Here, we first noticed that hLIR1 and hLIR2 are not conserved, indicating no conservation of functional residues. Human LIR3 and LIR4, on the other hand, display a strong conservation of the functional amino acids. However, the tyrosine in hLIR3 (^49^YAGL^52^) was replaced by a histidine residue in the *Sauropsida*, while the remaining amino acids of the LIR are conserved. Within the *Actinopterygii*, on the other hand, the leucine residue was replaced by glutamine. Human LIR4 exhibits frequent alterations in its X_2_ and Γ positions, while hLIR5 (^81^WVFV^84^) illustrates conservation throughout all *Deuterostomia* with only rare substitutions of valine to isoleucine in its X_1_ position (Figure 2B). Interestingly, this sequence features all characteristics of the GABARAP interaction motif consensus sequence [W/F]_Θ_ -[V/I]_1_-X_2_-V_Γ_,^50^ including the already mentioned flanking by N-terminal acidic residues. Human LIR6 to LIR9 likewise present a strong conservation of the amino acids required for an ATG8 protein interaction. Based on the phylogenetic conservation and under the premise that σ1R’s effect on autophagy is conserved as we have described for *C. elegans*,^38^ we would anticipate hLIR4 to hLIR9 to interact with ATG8 proteins, with hLIR5 being the most likely candidate based on its total conservation.

To further narrow down potential interaction sites, we looked at the structural accessibility of these motifs (Figure 2C). The two N-terminal motifs (hLIR1 and hLIR2) are located within the transmembrane helix (α1) and, therefore, are probably not accessible in the physiological membrane-bound form of σ1R. At the inactive state, human LIR3 and LIR4, located on the opposing side (α2 and α3, respectively) and probably facing the ER lumen,^58^ are potentially accessible. Likewise, hLIR5, located within the first beta sheet (β1), might also face the ER lumen. However, according to the trimeric organization,^11^ this motif is likely accessible in a monomeric state (Figure 2D). Binding of a ligand to the receptor and its subsequent activation, i.e., dissociation, could possibly regulate accessibility of this motif. Human LIR6 (β3), located within a crucial region for the binding of small molecule ligands,^59^ and hLIR7 (β5) are both structurally buried in the core of σ1R. Accordingly, these two motifs are likely not accessible for protein-protein interactions. Finally, hLIR8 and hLIR9 are both part of the most C-terminal helix (α5) and thus could be accessible.

Collectively, the conservation, phylogenetic, and structural analyses clearly point toward hLIR5 as the most likely interaction motif of σ1R for ATG8 proteins.

### σ1R hLIR5 interacts with GABARAP

**Figure 3:**
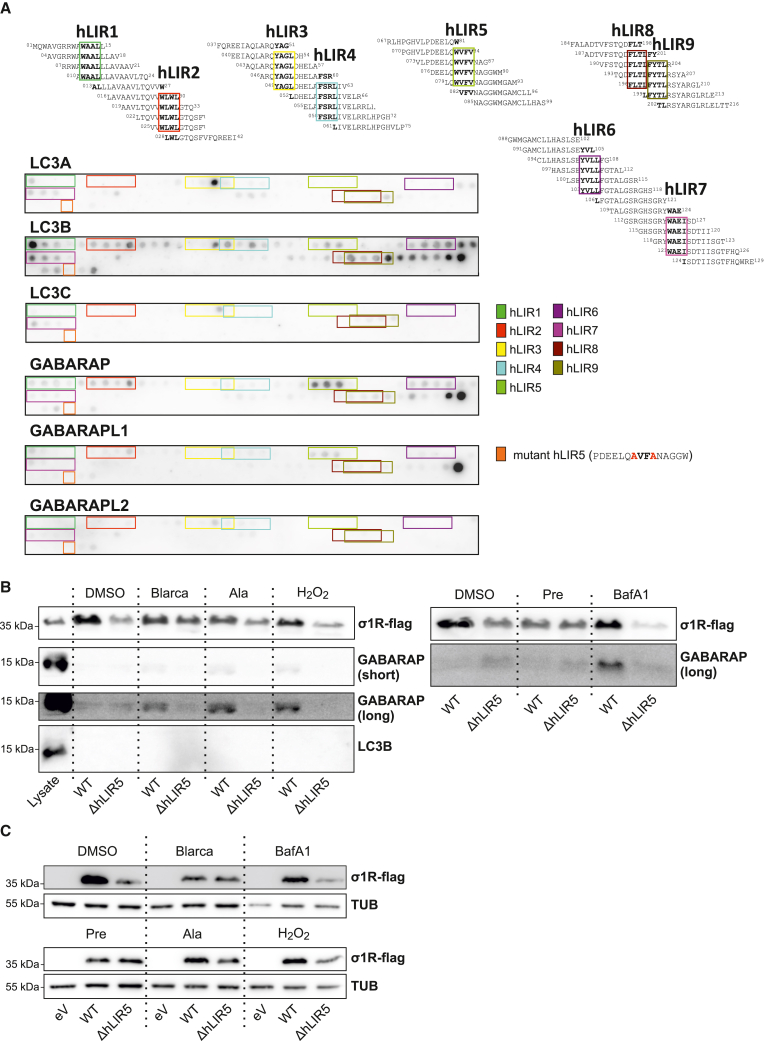
Biochemical assessment of human σ1R LIR motifs (A) Peptide array using spotted oligopeptides of σ1R (15mers with three amino acid offset) after incubation with recombinant GST-tagged LC3A, LC3B, LC3C, GABARAP, GABARAPL1, or GABARAPL2. Colored boxes represent peptides harboring putative LIR motifs. Negative control for hLIR5 (mutant hLIR5, orange box) displays no interaction with GABARAP. Tables display all peptides containing the respective LIR motif. (B) One representative of at least three independent Western blots of GABARAP, LC3B, and σ1R following immunoprecipitation with anti-FLAG-antibodies from HeLa cells transfected with FLAG-tagged wild type σ1R (WT) or a ΔhLIR5 construct and treated with σ1R activating compounds. (C) One representative of three independent Western blots of total lysates for immunoprecipitations in B. Treatment parameters (B and C): alazocine (Ala, 2.5 μM, 2h), bafilomyin A1 (BafA1, 2 μM, 2h), blarcamesine (Blarca, 10 μM, 2h), H_2_O_2_ (50 μM, 2h), PRE-084 (Pre, 10 μM, 2h).

To biochemically assess the binding properties of the *in silico* identified nine putative hLIR motifs, we performed a peptide array analysis and thereby screened the entire σ1R sequence for interactions with all human GST-tagged ATG8 proteins (Figure 3A). Signals are considered as positive interactions when at least three spots in a row were recognized within one putative LIR core motif. Hereby, we confirmed prominent interactions of LC3B and GABARAP with different σ1R hLIR motifs, whereas the other ATG8 proteins showed rather weak or hLIR-unrelated signals. Among those, only LC3A’s and LC3C’s interaction with hLIR7 peptides and LC3C’s interaction with hLIR9 peptides stand out slightly. Since hLIR7 is unlikely to be accessible for protein-protein interaction in the proper folded σ1R, we did not address this motif in subsequent analyses. LC3B and GABARAP display a largely overlapping interaction pattern with hLIR motifs. Notably, GABARAP, but not GABARAPL1 and GABARAPL2, prominently recognize hLIR5 (^81^WVFV^84^). This is consistent with the above-mentioned general motif specificity of ATG8 proteins (Table 1). In addition, the loss of the GABARAP signal with the peptide lacking the N-terminally located acidic residues (X_-5_-X_-3_) (Figure 3A; right peptide spot within the indicated hLIR5 region) indicated the importance of the N-terminal acidic residues for the affinity to the core motif.

To verify the significance of these so far exclusively bioinformatical and biochemical data on the cellular level, we decided to investigate the ATG8 proteins LC3B and GABARAP, which prominently interacted with σ1R in the peptide array, in more detail via co-immunoprecipitation analyses. Due to generally low endogenous σ1R expression levels in HeLa cells and unsatisfactory purification results with σ1R-specific antibodies, we performed the experiments in HeLa cells transiently overexpressing Myc-FLAG-tagged σ1R. In addition to basal conditions, we tested blarcamesine, an allosteric σ1R agonist that increases autophagic activity without increasing the quantity of autophagosomes,^38^ alazocine, a benzomorphan-based agonist inducing monomerization, and H_2_O_2_, an unspecific inducer of monomerization. Our immunoprecipitation assay did not confirm any interaction of LC3B with σ1R. LC3B neither co-precipitated with σ1R under basal conditions nor upon σ1R activation with blarcamesine, alazocine or H_2_O_2_ treatment (Figures 3B and 3C). However, GABARAP co-precipitated with σ1R upon blarcamesine, alazocine and H_2_O_2_ treatments, but hardly under basal conditions. To validate the specificity of the interaction of σ1R with GABARAP, we generated a σ1R variant with a mutation in the hLIR5 motif (ΔhLIR5) in which the tryptophane residue, one of the core amino acids responsible for the interaction with ATG8 proteins, is mutated into alanine (W81A, resulting in ^81^AVFV^84^).^48^ Interestingly, after blarcamesine, alazocine or H_2_O_2_ treatments the mutant hLIR5 variant co-precipitated endogenous GABARAP to a considerably lesser extent (Figure 3B).

**Figure 4:**
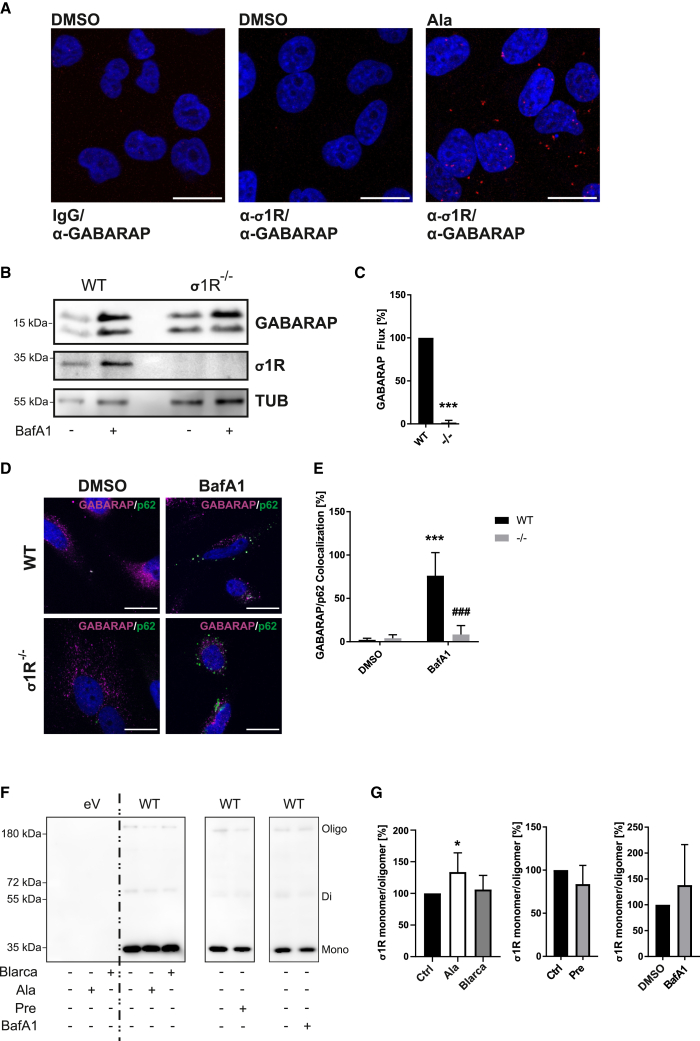
Evaluation of σ1R GABARAP interaction and merization state (A) Representative confocal images of untransfected HeLa cells obtained from three independent proximity ligation assays (PLA) of the interaction of endogenous σ1R and GABARAP (red), DAPI (blue), Magnification: 40×. Scale bar: 20 μm. (B) One representative of three independent Western blots of total lysates of GABARAP, σ1R, and tubulin (Tub) from wild type and σ1R-deficient (σ1R^−/−^) HeLa cells. (C) Statistical analysis of B by two-way ANOVA. Post hoc *p*-values were calculated using Benjamini–Hochberg. Statistics are depicted as mean ± SD of three independent experiments; ∗∗∗*p* ≤ 0.001. (D) Representative confocal images obtained from three independent immunofluorescence stainings of wild type and σ1R-deficient (σ1R^−/−^) HeLa cells, GABARAP signals are shown in magenta, p62 signals in green. Confocal images of single channels are depicted in Figure S1C. Magnification: 100×. Scale bar: 20 μm. (E) Quantification of proportion of p62 signals overlapping with GABARAP signals from D. Significance was statistically verified by two-way ANOVA. Post hoc *p*-values were calculated using Benjamini–Hochberg. Statistics are depicted as mean ± SD of three independent experiments with at least three randomly chosen optical fields containing at least three transfected cells; ∗∗∗*p* ≤ 0.001 between treatment, ###*p* ≤ 0.001 between wild type and σ1R^−/−^ cells. (F) One representative of at least three independent Western blots analyzing σ1R monomerization in HeLa cells transfected with FLAG-tagged wild type σ1R (WT) using an anti-FLAG-antibody antibody. A short exposure of the blot is depicted in Figure S1F. (G) Quantification of relative monomeric to oligomeric σ1R ratio from F. Statistics are depicted as mean ± SD, Significance was statistically verified by one-way ANOVA. Post hoc *p*-values were calculated using Benjamini–Hochberg. (A–G) Treatment parameters: alazocine (Ala, 2.5 μM, 2h), bafilomyin A1 (BafA1, 2 μM, 2h), blarcamesine (Blarca, 10 μM, 2h), PRE-084 (Pre, 10 μM, 2h).

In addition, we have also tested the compound PRE-084, an agonist which hardly appears to induce monomerisation,^20^ as well as bafilomycin A_1_ (BafA1). BafA1 inhibits the acidification of lysosomes and thus results in an increase of the number of autophagosomes as well as an accumulation of autophagy cargo/client proteins as a consequence of disabled degradation. We did not observe any increase in the interaction between σ1R and GABARAP under PRE-084 treatment. However, BafA1 treatment slightly induced this interaction (Figure 3B). Although we observed a variation in the respective expression levels, this effect still persists after normalization to the respective precipitated σ1R intensities. A possible explanation of the co-immunoprecipitation might be that monomerization is required for a hLIR5-dependent interaction with GABARAP. Even though co-precipitated GABARAP was increased upon both σ1R monomerization and BafA1 treatment, the increase upon BafA1 treatment might be attributed to the increased number of autophagosomes, or interactions within. This aligns with the observation that BafA1 is the only treatment that has an effect on GABARAP levels (Figure S1A). To analyze whether this interaction likewise occurs *in situ* in the cellular context, between the endogenous proteins, and whether it is induced via σ1R activation, as suggested by the *in silico* and co-immunoprecipitation results, we applied a proximity ligation assay (PLA). Here we also employed alazocine, a σ1R agonist that induces monomerization (Figure 4A). Confirming the immunoprecipitation data, we hardly detected a proximity signal of endogenous GABARAP with σ1R in the DMSO control. However, we observed a clear signal under alazocine treatment, suggesting an induction of this interaction (Figure 4A). In a following step, we generated σ1R-deficient HeLa cells to assess whether a loss of σ1R interaction affects autophagy, particularly GABARAP function. Indeed, the GABARAP flux in the σ1R-deficient cells completely collapsed (Figures 4B and 4C). Accordingly, this significantly reduced lipidation of GABARAP resulted in GABARAP-depleted autophagic vesicles, as reflected in loss of co-localization in confocal microscopy of p62 and GABARAP particularly under BafA1 treatment (Figures 4D, 4E, and S1C). The immunofluorescence results thus indicate a functional aspect of the immunoprecipitation results.

To further investigate whether monomerization is a prerequisite for the GABARAP interaction, we analyzed the merization status of σ1R^20^ for selected treatments. For the validation of the assay and induction of monomerization, we used alazocine. Accordingly, we observed a significant increase in the relative amount of monomeric σ1R. For blarcamesine treatment, we observed a tendency but no significant increase in monomeric σ1R, also not for PRE-084, which is consistent with the literature^20^ (Figures 4F and 4G). Due to the high variability of the results, we are not able to conclude any effect of BafA1 on monomerization.

Since not all agonists impact the monomeric to oligomeric σ1R ratio, whose complexity of activation was already specified in the introduction, we cannot definitively attribute the dependence of the GABARAP interaction to monomerization thus far. In the case of BafA1, the suspected induction might also be due to sheer accumulation in the absence of autophagosome clearance.

**Figure 5:**
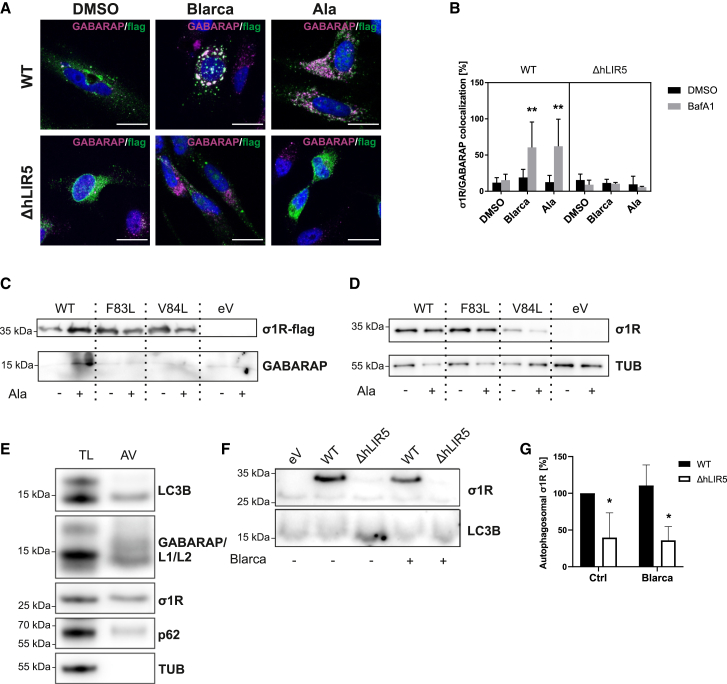
Induction of σ1R GABARAP co-localization, clinical mutations in hLIR5, and autophagic vesicle analysis (A) Representative confocal images obtained from three independent immunofluorescence stainings of HeLa cells transfected with FLAG-tagged wild type σ1R (WT) or a ΔhLIR5 construct and treated with BafA1. Confocal images of DMSO control and single channels are depicted in Figures S1D and S1E. GABARAP signals are shown in magenta, FLAG signals in green; Magnification: 100×. Scale bar: 20 μm. (B) Quantification of GABARAP signals overlapping with FLAG signals normalized to total FLAG signals from A. Significance was statistically verified by three-way-ANOVA. Post hoc *p*-values were calculated using Benjamini–Hochberg. Statistics are depicted as mean ± SD of three independent experiments with at least three randomly chosen optical fields containing at least three transfected cells. (C) One representative of three independent Western blots of GABARAP and σ1R following immunoprecipitation with anti-FLAG-antibodies from HeLa cells transfected with FLAG-tagged wild type σ1R (WT) or F83L and V84L mutants treated with alazocine. (D) One representative of three independent Western blots of total lysates for immunoprecipitations in C. (E) Representative Western blot (out of three independent experiments) for LC3B, GABARAP, σ1R, SQSTM1 (p62), and Tubulin (TUB) of total lysates (TL) (10 μg) compared with isolated autophagic vesicles (AV) (2∗10^6^ vesicles) from HeLa cells treated with BafA1 (2 μM, 2h). (F) Representative Western blots for σ1R and LC3B from isolated autophagic vesicles (2∗10^6^) from HeLa cells transfected with wild type σ1R (WT) or a ΔhLIR5 construct, all cells were treated with BafA1 (2 μM, 2h) and with or without blarcamesine (Blarca, 10 μM, 2h); samples loaded were normalized to the numbers of FACS-sorted autophagic vesicles. (G) Quantification of autophagosomal σ1R in F. Significance was statistically verified by two-way ANOVA. Calculated *p*-values (Holm-Sidak post hoc test) are displayed above the compared column. Statistics are depicted as mean ± SD of three independent experiments. (A–G) Treatment parameters: alazocine (Ala, 2.5 μM, 2h), bafilomyin A1 (BafA1, 2 μM, 2h), blarcamesine (Blarca, 10 μM, 2h).

To assess if the interaction with GABARAP is mediated via hLIR5, we investigated the co-localization of wild type σ1R and ΔhLIR5 σ1R under different treatments. Similar to the experiments described so far (Figures 3B and 4A), the co-localization experiments also indicated a low co-localization for wild type σ1R under basal conditions and increased interaction in response to treatment with σ1R agonists (Figure 5A). The ΔhLIR σ1R features hardly any co-localization with GABARAP, even after stimulation with σ1R agonists (Figure 5B). This additionally supports the rationale that the interaction of σ1R and GABARAP is indeed specific and facilitated by σ1R’s hLIR5 motif.

The clinical significance of the hLIR5 motif is highlighted by two known point mutations within this sequence, which are associated with ALS-mimicking or autosomal-recessive distal spinal muscular atrophy 2^60^ and NCBI ClinVar Database: VCV000579502.9, VCV000937637.7. Similar to Figure 3B, we performed immunoprecipitations of the two mutants (F83L and V84L) in comparison to wild-type σ1R. We found that both σ1R mutants were no longer able to co-precipitate GABARAP even upon stimulation with alazocine (Figures 5C and 5D).

### hLIR5 links σ1R to autophagic vesicles

As the analysis of our confocal images suggests that σ1R and GABARAP co-localize at vesicular structures (Figures 4D and 5A), we biochemically analyzed isolated native autophagic vesicles to gain deeper insights into whether the hLIR5-mediated interaction links σ1R to autophagic vesicles. Thereto, we applied a recently established purification protocol to isolate native autophagic vesicles from cultured cells by a combination of successive centrifugation steps and subsequent sorting of antibody-based fluorescence-tagged ATG8-positive structures via flow cytometry.^61^ This is reflected in the depletion of cytoplasmic components such as tubulin with the concomitant accumulation of lipidated autophagosome-associated LC3B-II, GABARAP proteins, and the autophagy receptor SQSTM1/p62 (Figure 5E). This is in line with previous isolations attained with the published protocol.^61^ In this assay, we routinely applied BafA1 treatment to maximize the amount of autophagosomal material, which in this case already increased the level of σ1R-GABARAP interaction (Figures 5F and 5G). For immunoblot quantification, we analyzed 2∗10^6^ autophagic vesicles each by normalizing to the initial amount of tubulin and expression level of wild type σ1R and ΔhLIR5, respectively. The analysis of the purified autophagic vesicles clearly confirms the presence of σ1R in these structures (Figure 5G). Strikingly, there is a clear indication that the autophagic vesicle-association of σ1R is abrogated by the single point mutation (ΔhLIR5) (Figures 5F and 5G). It is of note that additional blarcamesine treatment on top of the BafA1 treatment failed to significantly increase the autophagosomal amount of wild type or ΔhLIR5 σ1R (Figure 5G).

## Discussion

Our study provides evidence for a LIR-specific interaction between the σ1R and the ATG8 protein GABARAP that is inducible through ligand-dependent σ1R activation. This finding directly links the two proteins, which are both functionally attributed to autophagosome-lysosome fusion.^37^^,^^45^^,^^46^ The motif mediating this interaction (hLIR5) is present and potentially functional in all *Craniota* (Figure 2B). Here, the comparably strong conservation of the N-terminally located acidic residues (X_-5_-X_-3_) emphasizes their significance for binding to this motif. Accordingly, ^75^DEELQWVFV^84^ appears to be the probable core LIR sequence required for σ1R to interact with GABARAP, as also indicated by the peptide array (Figure 3A).

Both GABARAP and σ1R have already evolved in *Placozoa*, one of the simplest multicellular organisms. Strikingly, the orthologous sequence to hLIR5, although lost in *Phallusia* *sp.*, actually exists in the *Placozoa Trichoplax* *sp.* (^75^WIFI^78^ including two N-terminally located acidic residues) and therefore may feature potential functionality regarding ATG8 binding. As the overall σ1R sequence is strongly conserved, with at least 60% sequence identity in all *Craniota*—it even features nearly 30% identity with the C8-C7 sterol isomerase ERG2 from yeast^62^— the conservation of hLIR6 and hLIR7 might also be attributed to the significance of ligand binding which is located in this region.^59^ Therefore, we assume that the identified binding sites (hLIR6 and hLIR7) of the σ1R peptides with LC3B in the peptide array are not accessible in the properly folded protein, which is supported by the failure to co-precipitate LC3B (Figures 2C and 3B). Consistently, we assume that, in terms of cell physiology, hLIR9 is also not relevant for LC3B interaction at the protein level.

However, for the GABARAP-hLIR5 interaction, both the structural accessibility (Figure 2C) of the motif and monomerization of σ1R (Figures 3B, 4A, and 5A) suggest a physiological regulation of this interaction via the activation of σ1R. This is also supported by the results of the PLA. Only receptor stimulation induces an interaction within the cell.

We would like to highlight that activation of σ1R by PRE-084,^20^ was not sufficient to induce this interaction (Figure 3B). Furthermore, we were not able to demonstrate a monomerization effect by blacarmesine treatment. Likewise, treatment with BafA1 also induced the interaction in immunoprecipitation, but also had no direct effect on monomerization (Figure 4F). Here, however, the accumulation of autophagosomes might cause this effect (Figures 5F and 5G). Thus, our data (Figure 3B) suggest that monomerization, as induced by alazocine and H_2_O_2_, enables GABARAP binding to σ1R. To become accessible to cytoplasmic or autophagosomal GABARAP, σ1R requires, besides monomerization, at least a partial translocation caused by activation.^6^^,^^8^ Similarly, the PLA suggests that activation and probably induced translocation enable interaction with GABARAP. However, due to the considerable effect on the lipidation and autophagosomal localization of GABARAP caused by σ1R deficiency, we expect a significant impact on autophagy.

Although the mechanism is not yet fully understood, we assume that a loss of autophagosomal GABARAP could impair autophagosome-lysosome fusion.^37^ Similarly, in the course of this fusion mechanism, σ1R interacts with syntaxin-17, VAMP8, and ATG14.^37^ As all of these proteins are exclusively present on the surface of autophagosomes or lysosomes, we assume that σ1Rs LIR-dependent interaction also involves surface bound GABARAP. However, we cannot exclude the possibility of σ1R binding to GABARAP also inside of autophagosomes. It also remains elusive whether σ1R specifically binds lipidated or unlipidated GABARAP. Precise characterization of the actual localization of σ1R at or within autophagosomes would provide a clear indication of its function in this context. A surface association may influence GABARAP function in autophagosome-lysosome fusion,^45^^,^^46^ whereas a LIR-dependent localization within the vesicle may also argue for a function as a cargo receptor.

In conclusion, our study connects the autophagy modulating protein σ1R with the autophagic ATG8 system via *in silico*, biochemical, and cellular methods and indicates a direct interaction between σ1R and GABARAP mediated by one functional LIR motif, hLIR5. The relevance of hLIR5 is highlighted by its conservation throughout the *Metazoa*. Notably, both known human mutations within hLIR5 (F83L and V84L) are linked to ALS-mimicking or autosomal-recessive distal spinal muscular atrophy 2, which further underlines the relevance of hLIR5. Given both mutants no longer co-precipitated GABARAP, our data hint toward a potential pathobiochemical significance of these findings. The interaction reported in this study could represent a missing biochemical link in autophagy modulation by σ1R.

### Limitations of the study

Our study evaluates the interaction of σ1R with ATG8 proteins. The binding was analyzed *in silico*, biochemically *in vitro,* and *in situ* in HeLa cells. Cell biologically, the induction of interaction was examined exclusively in immortalized cancer cells (wild-type and σ1R knock-out) with the overexpression of σ1R. Future studies need to elucidate the precise structure of the σ1R -GABARAP complex and address the physiological implications and regulation of this interaction at the organismic level.

## Resource availability

### Lead contact

Requests for further information and resources should be directed to and will be fulfilled by the lead contact, Hagen Körschgen (hagen.koerschgen@uni-mainz.de).

### Materials availability

All materials generated in this study are available from the lead contact upon reasonable request.

### Data and code availability

• *Data*: All data reported in this article will be shared by the lead contact upon reasonable request.
• *Code*: This study does not report original code.
• *Other items*: Any additional information and raw data will be shared by the lead contact upon reasonable request.

## Acknowledgments

The compound blarcamesine was kindly provided by Anavex Life Sciences Corporation. The authors thank Fazilet Bekbulat (Institute of Pathobiochemistry, UMC Mainz, Germany) for the critical reading of the article.

This work was supported by the Deutsche Forschungsgemeinschaft (DFG, German Research Foundation) Project-ID 259130777-SFB 1177, and Project-ID 530063157 (ANR/DFG), by the Corona Foundation of the Stifterverband für die deutsche Wissenschaft, and by the Hanna-Bragard-Apfel Foundation.

## Author contributions

Substantial contributions to conception and design (CB, HK, MWB, and MC). Acquisition of data, or analysis and interpretation of data (DS, HK, HN, MWB, MC, AMC, and WT). Drafting the article or revising it critically for important intellectual content (CB, HK, MWB, MC, and AMC). Final approval of the version to be published (all authors).

## Declaration of interests

The authors declare no competing interests.

## STAR★Methods

### Key resources table

**Table:** 

REAGENT or RESOURCE	SOURCE	IDENTIFIER
**Antibodies**		

Anti-GST	GE Healthcare	RPN1236
Anti-GABARAP	MBL	M135-3
Anti-LC3B	Sigma-Aldrich	L7543
Anti-p62	Progen	GP62-C
Anti-TUB	Serotec	MCA P77
Anti- σ1R	Thermo Fischer Scientific	42-3300
Anti-FLAG®	Sigma-Aldrich	F1804; RRID:AB_262044
Anti-GABARAP/GABARAPL1/GABARAPL2	Abcam	ab223948
Cy 2 Anti-Mouse	Jackson ImmunoResearch	715-225-151; RRID:AB_2340827
Cy 3 Anti-Rabbit	Jackson ImmunoResearch	711-165-152; RRID:AB_2307443
Cy5 Anti Guinea pig	Jackson ImmunoResearch	706-175-148; RRID:AB_2340462
HRP anti guinea pig	Jackson ImmunoResearch	706-035-148; RRID:AB_2340447
HRP anti rabbit	Jackson ImmunoResearch	711-035-152; RRID:AB_10015282
HRP anti mouse	Jackson ImmunoResearch	715-035-151; RRID:AB_2340771

**Chemicals, peptides, and recombinant proteins**		

GST-LC3A	NovusBio	H00084557-P01
GST-LC3B	Novoprolabs	509467
GST-LC3C	Abnova	H00440738-P01
GST-GABARAP	Abnova	H00011337-P01
GST-GABARAPL1	Biomol	373.380.100
GST-GABARAPL2	Abnova	H00011345-Q01
Pre-084	Tocris Life Sciences	0589
Alacozine	Tocris Life Sciences	1079
H_2_O_2_	Sigma-Aldrich	H1009
Bafilomycin A1	Toronto Research Chemicals	B110000
TransIT-293 Transfection Reagent	Mirus Bio	2700
DMEM	Invitrogen	41965062
Fetal bovine serum	Life Technologies	10270106
Antibiotic-antimycotic solution	Invitrogen	15240-062
Sodium pyruvate	Invitrogen	1136-088
Blarcamesine	ANAVEX Life Sciences	ANAVEX® 2-73

**Critical commercial assays**		

QuikChange Lightning Site-Directed Mutagenesis Kit	Agilent	210518
Duolink® proximity ligation assay	Merck	DUO920082, DUO92004, DUO92008

**Experimental models: Cell lines**		

HeLa	Körschgen et al.^54^	CVCL_2506

**Oligonucleotides**		

gcattcacgaacaccgcctgcagctcctcgtc	Eurofins	dLIR_for
gacgaggagctgcaggcggtgttcgtgaatgc	Eurofins	dLIR_rev
CCCACTGCAGCTCCTCGTC	Merck	gRNA1
CAGCACCGCTGCGACAGCC	Merck	gRNA2

**Software and algorithms**		

ClustalO		SCR_001591
iTOL v6		Letunic, and Bork^63^
Skylign		Wheeler et al.^64^
Fiji		SCR_002285
COLABFOLD v1.5.2		Mirdita et al.^57^
FlowJo v10.6.1	BD Biosciences	SCR_008520
UCSF CHIMERA	UCSF	SCR_004097
GraphPad Prism	GraphPad	SCR_002798
iLIR		https://ilir.warwick.ac.uk/

**Other**		

SIGMAR1 Myc- FLAG®	OriGene	RC201206
SIGMAR1F83L Myc- FLAG®	VectorBuilder	N/A
SIGMAR1V84L Myc- FLAG®	VectorBuilder	N/A
MISSION™ gRNA LV01 U6-gRNA:EF1a-puro-2A-Cas9-2A-tGFP	Merck	N/A
Anti-FLAG® M2 magnetic beads	Merck	M8823
LSM710	Zeiss	SCR_018063
Spotted peptide membranes	Intavis Peptides Services	N/A

### Experimental model and study participant details

In this study we only used HeLa cells which were initially authenticated via STR profiling and regularly tested for mycoplasma contamination. Cells were cultured in DMEM (Invitrogen, 41965062) supplemented with active FBS (Life Technologies, 10270106), ABAM (Invitrogen, 15240-062) and 1 mM sodium pyruvate (Invitrogen, 1136-088). Cultures were kept at 37°C in a humidified atmosphere containing 5% CO2. For transient overexpression, HeLa cells were transfected with SIGMAR1 Myc- FLAG®-tagged plasmid (OriGene, RC201206) via calcium phosphate precipitation. A single point LIR mutant (σ1R ΔhLIR5) was generated using the QuikChange Lightning Site-Directed Mutagenesis Kit (Agilent, 210518) (Primers: 5'-gcattcacgaacaccgcctgcagctcctcgtc-3' and 5'-gacgaggagctgcaggcggtgttcgtgaatgc-3') according to the manufacturer's instructions. Successful mutagenesis was confirmed via Sanger sequencing. For the F83 and V84L experiments, the wild type and mutants of the Myc- FLAG®-tagged SIGMAR1 construct were ordered from VectorBuilder (Neu-Isenburg, Germany). 48 h post transfection, cells were treated for 2 h with 10 μM blarcamesine (ANAVEX® 2-73; provided by ANAVEX Life Sciences),^38^ 10 μM PRE-084 (Tocris Bioscience, 0589),^38^ 2.5 μM alazocine (SK&F 10047, Tocris Biocience; 1079),^20^^,^^65^ 50 μM H_2_O_2_ (Sigma-Aldrich, H1009)^20^ and/or 2 μM bafilomycin A1 (Toronto Research Chemicals, B110000)^61^ or left untreated as control. For monomerization assays, HeLa cells were transfected using TransIT-293 Transfection Reagent (Mirus Bio, 2700) according to the manufacturer’s instructions. After 24 h cells were treated as indicated above.

### Method details

#### Sequence alignments & phylogenetic tree

Protein sequences were obtained from NCBI’s nucleotide database and UniProt. We aligned sequences using the ClustalO software.^66^ The phylogenetic tree was visualized using the iTol v6 software.^63^ LIR sequence logos were created using Skylign.^64^ Putative human LIR sequences were obtained using the webtool iLIR (https://ilir.warwick.ac.uk/).

#### In silico structure analysis

We created models of the monomeric human σ1R using COLABFOLD v1.5.2 (https://colab.research.google.com) based on ALPHAFOLD2.^56^^,^^57^ The best ranked model was used for further analysis. Models were visualized using UCSF CHIMERA.^67^^,^^68^

#### Peptide array

The membranes with spotted 15mer peptides and an offset of three amino acids of σ1R were obtained from Intavis Peptides Services (Tübingen, Germany). The Peptide array was carried out following the manufacturer’s protocol and incubated with heterologously expressed GST-tagged LC3s (LC3A, Novusbio, H00084557-P01; LC3B, Novoprolabs, 509467; LC3C, Abnova, H00440738-P01) or GABARAPs (GABARAP, Abnova, H00011337-P01; GABARAPL1, Biomol, 373.380.100; GABARAPL2, Abnova, H00011345-Q01) at 2 μg/ml. Binding of the proteins was evaluated using an anti-GST-HRP conjugate (GE Healthcare, RPN1236).

#### Immunoprecipitation

Cells were lysed in immunoprecipitation buffer (50 mM Tris, pH 7.4, 150 mM NaCl, 2 mM Na2EDTA, 5% (v/v) glycerol, 0.5% (v/v) IGEPAL CA-630, 1 mg/ml cOmplete™ EDTA-free) and passed three times through a 23G needle. Protein concentration was determined using BCA assay (Thermo Fisher Scientific, 23225). The lysate (500 μg) was incubated with anti-FLAG® M2 magnetic beads (Merck, M8823) at 4 °C for 60 min. After three washing steps, elution was accomplished via 2x SDS loading buffer (125 mM Tris, 4% (w/v) SDS, 20% (v/v) glycerol, 50 mM dithiothreitol, pH6.8). Samples were separated via SDS-PAGE and analyzed via immunoblotting utilizing mouse anti-GABARAP (1:1000, MBL, M135-3), anti-LC3B (1.1000, Sigma-Aldrich, L7543), anti-FLAG® (1:1000, Sigma-Aldrich; F1804), anti-tubulin (1:1000, Serotec, MCA P77), guinea-pig anti-p62 (1:1000, Progen, GP62-C) and rabbit anti-σ1R (1:1000, Thermo Fisher Scientific, 42-3300).

#### Proximity ligation assay

HeLa cells were cultured in as described for immunostaining. Cells were fixed with formaldehyde and permeabilized using ice-cold 90% methanol. The sample preparation was conducted using Duolink® proximity ligation assay (PLA; Merck, DUO920082, DUO92004, DUO92008) according to the manufacturer's protocol with an amplification time of 150 min with a rabbit anti-σ1R (Thermo Fisher Scientific, 42-3300, 1:200) and a mouse anti-GABARAP (MBL International, M135-3, 1:200). Stained cells were imaged with a Zeiss LSM710 confocal microscope. All pictures were taken with a 40x objective (1024 × 1024 pixels).

#### Analysis of σ1R merization status

After treatment, cells were washed in 1x phosphate buffered saline (PBS) and centrifuged at 600 x g for 3 min. Then, cells were lysed in 100 μl modified radioimmunoprecipitation assay (RIPA) buffer (50 mM Tris-HCl, pH 7.4, 150 mM NaCl, 0.5 % (v/v) Triton X-100, 0.05 % (v/v) SDS, 0.05 % (v/v) sodium deoxycholate, 1 mg/ml cOmplete™ EDTA-free (Roche, 04693132001), 1x PhosSTOP™ (Roche, 04906845001)) and incubated on ice for 15 min. Lysates were centrifuged at 18 000 x g, 4 °C for 10 min and supernatant was collected. Protein concentration was determined using BCA assay (Thermo Fisher Scientific, 23225). 4x SDS loading buffer (278 mM Tris-HCl, pH 6.8, 40 % (v/v) glycerol, 4 % (v/v) SDS) with 10 % 2-mercaptoethanol was added to 30 μg of protein. Samples were not heated prior to separation via SDS-PAGE. Subsequently, samples were analyzed via immunoblotting utilizing mouse anti-FLAG® (1:1000, Sigma-Aldrich; F1804).

#### Generation of a σ1R-deficient HeLa cell line and GABARAP flux calculation

The CRISPR/Cas9 constructs were obtained from Merck as MISSION™ gRNA plasmids (MISSION™ gRNA LV01 U6-gRNA:EF1a-puro-2A-Cas9-2A-tGFP) with 5'-CCCACTGCAGCTCCTCGTC- 3' and 5'-CAGCACCGCTGCGACAGCC- 3' as guide RNA. To generate stable σ1R-deficient HeLa cells. Wild type cells were transfected using electroporation. Transfected cells were selected by puromycin and separated in a 96-well plate. Individual positive clones were expanded and checked for a successful knockout by Western blot analysis and qPCR. We calculated the GABARAP flux by analyzing the difference of the GABARAP signals in the Western blot of total lysate in the presence and absence of bafA1 treatment, normalized to tubulin, and determined the relative ratio between wild type and σ1R deficient cells.

#### Immunofluorescence staining of cells

HeLa cells were grown on glass cover slips, treated as indicated, and fixed with 4% formaldehyde. Unspecific epitopes were blocked with 3% (w/v) albumin before permeabilization with 0.1% (v/v) Triton X-100 in PBS. The fixed cells were then incubated overnight with primary antibodies diluted in PBS containing 1% albumin: rabbit anti-GABARAP (1:200, Cell Signaling, 13733) and mouse anti-FLAG® (1:500, Sigma-Aldrich; F1804). Subsequently, cells were incubated with Cy3- and Cy5-coupled secondary antibodies (1:500, Jackson Immunoresearch) for 2 h at room temperature and analyzed using a Zeiss LSM 710 confocal laser-scanning microscope. All pictures were taken with a 100x objective (1024 × 1024 pixels).

#### Autophagic vesicle purification

Autophagic vesicles were purified based on the method described previously.^61^ At least 1 x 107 cells were collected using Trypsin/EDTA and centrifuged at 306 x g for 4 min. After resuspension in PBS supplemented with cOmplete™ EDTA-free (Roche), cell disruption was performed using a UP50H ultrasonic processor (Hielscher) for 3 x 2 s with an amplitude of 60 %. Samples were then centrifuged at 3,000 x g for 10 min at 4 °C, supernatants were collected and centrifuged at 18,620 x g for 1 h at 4 °C. Pellets were washed and resuspended in PBS and subsequently incubated with 4 μg/ml of PE-conjugated GABARAP/GABARAPL1/GABARAPL2 antibody (Abcam, ab223948) for 1 h. The samples were centrifuged again with 18,620 x g for 1 h at 4 °C, pellets were washed and resuspended in PBS. For fluorescence-activated vesicle sorting, a BD FACSAria III SORP (BD Biosciences) equipped with a 70 μm nozzle and a 1.0 FSC neutral density filter was used. The compartment containing autophagic vesicles was first established using an FSC/SSC plot on a logarithmic scale, followed by a doublet discrimination gate using SSC-A/W. Autophagic vesicles were defined as PE-positive events (561 nm, BP 586/15), whose positivity was conducted according to the background given by an unstained negative control. Vesicle sorting was achieved using minimum speed (flow rate < 3.0) maintaining less than 19,000 events per second. Analysis was performed using FlowJo v10.6.1 (BD Biosciences). Proteins of isolated autophagic vesicles were analyzed using a methanol/chloroform (2:1) precipitation protocol and subsequent resuspension in urea buffer (8 M urea and 4 % (w/v) CHAPS in 30 mM Tris (pH 8.5 with HCl)), including EDTA-free protease inhibitor. For immunoblot quantification, we analyzed two million autophagic vesicles each by normalizing to the initial amount of tubulin and expression levels of wild type σ1R and hΔLIR5 σ1R, respectively.

### Quantification and statistical analysis

Western blot and microscopy results were quantified using Fiji imageJ.^69^ Statistics are depicted as mean ± SD from three biological replicates, significance was statistically verified by one-way ANOVA. Post hoc p-values were calculated using Benjamini–Hochberg using GraphPad Prism v8. For Figures 4E and 5G, two-way ANOVA was used instead, and for Figure 5B three-way ANOVA. Significance was established using an α level of 0.05 and visualization of significant differences for separate factors were distinguished using asterisks (∗) for variable 1 or hashtags (#) for variable 2.
