# Supplementary material for: Conserved LIR-specific interaction of Sigma-1 receptor and GABARAP
Source: iScience. 2025 Aug 5;28(9):113287. doi: 10.1016/j.isci.2025.113287 (PMC12398221; doi:10.1016/j.isci.2025.113287)
Supplement: Document S1. Figure S1 [file mmc1.pdf]

iScience, Volume *28*

Supplemental information

Conserved LIR-specific interaction of Sigma-1 receptor and GABARAP

Marius Wilhelm Baeken, Maximilian Christ, Daniel Schmitt, Wencke Trein, Heike Nagel, Albrecht Martin Clement, Hagen Körschgen, and Christian Behl

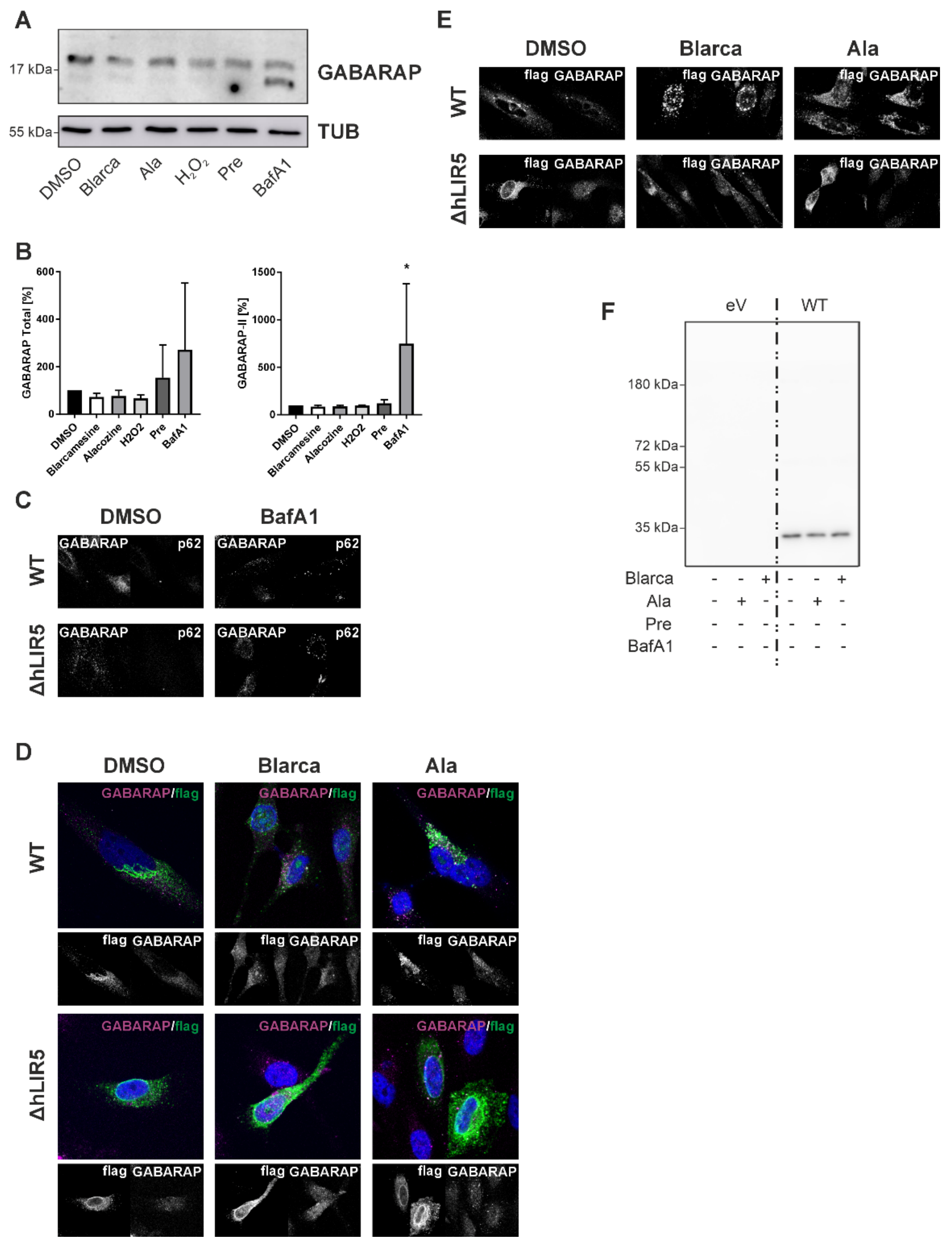

Supplemental Figure S1. Induction of σ1R GABARAP colocalization

(A) One representative of three independent Western blots of total lysates for immunoprecipitations. Treatment parameters: alazocine (Ala, 2.5 μM, 2h), bafilomyin A1 (BafA1, 2 μM, 2h), blarcamesine (Blarca, 10 μM, 2h).
(B) Statistical analysis of A by one-way-ANOVA. Post hoc p-values were calculated using Benjamini–Hochberg. Statistics are depicted as mean ± SD of three independent experiments; $*p \leq 0.05$.
(C) Single channels of merged depiction in Figure 4 D, Magnification: 100x. Scale bar: 20 μm.
(D) DMSO controls for BafA1 treated cells in Figure 5 A and statistically analyzed in Figure 5 B. Representative confocal images obtained from three independent immunofluorescence stainings of HeLa cells transfected with FLAG®-tagged wild type σ1R (WT) or a ΔhLIR5 constructs. GABARAP signals are shown in magenta, FLAG® signals in green; Magnification: 100x. Scale bar: 20 μm.
(E) Single channels of merged depiction in Figure 5 A, Magnification: 100x. Scale bar: 20 μm.
(F) Short exposure of the Western blot depicted in figure 4 F analyzing σ1R monomerization in Hela cells transfected with FLAG®-tagged wild type σ1R (WT) using an anti-FLAG®-antibody antibody.
